# Supplementary material for: Functional Characterization of the Lin28/let-7 Circuit During Forelimb Regeneration in Ambystoma mexicanum and Its Influence on Metabolic Reprogramming
Source: Front Cell Dev Biol. 2020 Nov 19;8:562940. doi: 10.3389/fcell.2020.562940 (PMC7710800; doi:10.3389/fcell.2020.562940)
Supplement: Supplementary file 1 [file Presentation_1.pdf]

Functional Characterization of the Lin28/let-7 Circuit during Forelimb Regeneration in *Ambystoma mexicanum* and its Influence on Metabolic Reprogramming

Hugo Varela-Rodríguez[1], Diana G. Abella-Quintana[1], Annie Espinal-Centeno[1], Luis Varela-Rodríguez[2], David Gomez-Zepeda[3], Juan Caballero-Pérez[1], Paola L. García-Medel[4], Luis G. Brieba[4], José J. Ordaz-Ortiz[3] and Alfredo Cruz-Ramirez[1, *].

[1] Molecular and Developmental Complexity Group, Unidad de Genómica Avanzada (LANGEBIO), Centro de Investigación y de Estudios Avanzados del IPN, Guanajuato, México.

[2] Facultad de Ciencias Químicas, Universidad Autónoma de Chihuahua, Chihuahua, México.

[3] Mass Spectrometry and Metabolomics Laboratory, Unidad de Genómica Avanzada (LANGEBIO), Centro de Investigación y de Estudios Avanzados del IPN, Guanajuato, México.

[4] Structural Biochemistry Group, Unidad de Genómica Avanzada (LANGEBIO), Centro de Investigación y de Estudios Avanzados del IPN, Guanajuato, México.

[*] Corresponding author: alfredo.cruz@cinvestav.mx (ACR).

SUPPLEMENTARY METHODS

In silico prediction of putative targets for the let-7 family of microRNAs

The 3'-UTR sequences of the previously reported axolotl transcriptome were used (Caballero-Pérez *et al.*, 2018). The prediction of let-7 targets was made using TargetScan v6.0 (Garcia *et al.*, 2011; Grimson *et al.*, 2007), and RNAhybrid v2.2 (Rehmsmeier *et al.*, 2004) under the parameters "-g all -D -n 22 -c -b 5 -G -f 2,8 -m 13153". Subsequently, a functional classification was performed with PANTHER v15.0 (Mi *et al.*, 2019) using the FISHER exact test and a False Discovery Rate correction to identify statistically overrepresented pathways, based on *Homo sapiens* gene list as reference. The results of the analysis are shown in Table S3.

Quantitative measurements for microscopy images

The intensity of the signal detected in the immunolocalizations made for amxLin28A and amxLin28B was quantified through Fiji/ImageJ v2.0/1.52i (Schindelin *et al.,* 2012), following the procedures performed in other studies with signal colocalization (Arqués *et al.,* 2012; Jonkman *et al.,* 2020). Cell

counts based on presence/absence of signal for amxLin28A and amxLin28B were performed with QuPath v0.2.2 (Bankhead *et al.*, 2017), adjusting the parameters for cell detection as follows: channel DAPI as reference; nuclear background radius 0 px; median filter radius 0 px; sigma 13 px; nuclear minimum area 10 px^2; nuclear maximum area 200,000 px^2; intensity threshold 10; split by shape; cell expansion 10 px; smooth boundaries; tile size 400 px trimmed to ROI (Mysona *et al.*, 2020). Statistical analyses were made with Minitab v16.1 (Minitab Inc.).

In silico prediction of secondary structures and 3'-UTR complementarity analysis for the *lin-28* family

In the complementarity analyzes between microRNAs members of the let-7 family and 3'-UTR target regions of the *lin-28* family, several 3'-UTR sequences used of representative organisms for the Tetrapoda superclass were obtained from the NCBI with the following accession numbers: *Homo sapiens: hsa-lin-28a* (XM_011542148.2) and *hsa-lin-28b* (NM_001004317.4); *Rattus norvegicus: rno-lin-28a* (NM_001109269.1) and *rno-lin-28b* (XM_001069344.5); *Monodelphis domestica: mdo-lin-28a* (XM_001363432.2) and *mdo-lin-28b* (XM_007484374.2); *Phascolarctos cinereus: pci-lin-28a* (NW_018343966.1) and *pci-lin-28b* (NW_018344024.1); *Cuculus canorus: ccn-lin-28a* (NW_009245496.1) and *ccn-lin-28b* (NW_009245766.1); *Parus major: pmj-lin-28a* (NW_015379252.1) and *pmj-lin-28b* (XM_015623265.3); *Meleagris gallopavo: mga-lin-28a* (NC_015035.2) and *mga-lin-28b* (XM_010707561.3); *Gallus gallus: gga-lin-28a* (NW_020109710.1) and *gga-lin-28b* (NC_006090.5); *Apteryx rowi: aro-lin-28a* (NW_020448197.1) and *aro-lin-28b* (NW_020448642.1); *Chrysemys picta: cpi-lin-28a* (NW_007281401.1) and *cpi-lin-28b* (NW_007359900.1); *Chelonia mydas: cmy-lin-28a* (NW_006648290.1) and *cmy-lin-28b* (NW_006660951.1); *Alligator mississippiensis: ami-lin-28a* (NW_017710296.1) and *ami-lin-28b* (NW_017713446.1); *Protobothrops mucrosquamatus: pmc-lin-28a* (NW_015387879.1) and *pmc-lin-28b* (NW_015386134.1); *Pseudonaja textilis: ptx-lin-28a* (NW_020769327.1) and *ptx-lin-28b* (NW_020769312.1); *Gekko japonicus: gja-lin-28a* (NW_015165859.1) and *gja-lin-28b* (NW_015177024.1); *Ambystoma mexicanum: amx-lin-28a* (MN268576.1) and *amx-lin-28b* (CM010933.1); *Pleurodeles waltl: pwa-lin-28a/b* (Matsunami *et al.*, 2019); *Nanorana parkeri: npa-lin-28a* (NW_017308326.1) and *npa-lin-28b* (NW_017306391.1); *Xenopus tropicalis: xtr-lin-28a* (NC_030678.2) and *xtr-lin-28b* (NC_030681.2); *Xenopus laevis: xla-lin-28a* (NC_030727.1) and *xla-lin-28b* (NC_030733.1); *Rhinatrema bivittatum: rbi-lin-28a* (NC_042625.1) and *rbi-lin-28b* (NC_042617.1). The context score results were generated with TargetScan v6.0 (Garcia *et al.*, 2011;

Grimson *et al.*, 2007) and graphed using IBS v1.0.3 107 (Liu *et al.*, 2015). Sequence alignments were performed with MAFFT v7.017 (Katoh *et al.*, 2002) in Auto mode. The folding of RNA secondary structures was performed with RNAfold v2.4.8 (Mathews *et al.*, 1999) under the parameters "-p -d 2 --noLP --noGU -P rna_turner1999.par" and adjusting the temperature (-T) to 37 for *H. sapiens,* or 20 for *A. mexicanum* sequences.

Western blot analysis

Total protein was extracted from wild-type juvenile axolotls. Tissue samples were homogenized in RIPA buffer (50 mM Tris, pH 8, 150 mM sodium chloride, 1% Nonidet P-40, 0.5% sodium desoxycholat, 0.1% sodium dodecyl sulfate, 1 mM phenylmethylsulfonyl fluoride, 4 µg/mL aprotinin, 1mM EDTA) at 4°C. After, samples were sonicated 10 times for 10 seconds, with intervals of 40 seconds. The samples were centrifuged at 14,500 g for 20 min at 4°C. For denaturation, we boiled the samples, with 2X Lamemmli buffer, for 3 min prior running in SDS-polyacrylamide electrophoresis gel at 180 V. After we transferred the gel to a PVDF membrane at 20 V for 30 min. The membranes were blocked in 5% nonfat dried milk for 2 h at 4°C. After the membranes were incubated overnight in the primary antibody solution at 4°C, for Anti-Lin28A (abcam, ab170402) was used a 1:200 dilution, while for Anti-Lin28B (Atlas Antibodies, HPA061745) a 1:500 dilution. After, we incubated membranes in the secondary antibody solution with Anti-Rabbit IgG Alkaline Phosphatase (AP) dilution 1:30,000 (Sigma, A3687) for 2 h at 4 °C. The membranes were then washed with a TBST buffer and incubated with AP substrate solution to reveal the signal.

REFERENCES

Arqués, O., Chicote, I., Tenbaum, S. P., Puig, I., Pálmer, H. G. (2012). Standardized Relative Quantification of Immunofluorescence Tissue Staining. *Protocol Exchange* protocol version 1. doi: 10.1038/protex.2012.008

Bankhead, P., Loughrey, M. B., Fernández, J. A., Dombrowski, Y., McArt, D. G., Dunne, P. D., McQuaid, S., Gray, R. T., Murray, L. J., Coleman, H. G., James, J. A., Salto-Tellez, M., & Hamilton, P. W. (2017). QuPath: Open source software for digital pathology image analysis. *Sci. Rep.* 7, 16878. doi: 10.1038/s41598-017-17204-5

Caballero-Pérez, J., Espinal-Centeno, A., Falcon, F., García-Ortega, L. F., Curiel-Quesada, E., Cruz-Hernández, A., Bako, L., Chen, X., Martínez, O., Alberto Arteaga-Vázquez, M., Herrera-

Estrella, L., & Cruz-Ramírez, A. (2018). Transcriptional landscapes of Axolotl (Ambystoma mexicanum). *Dev. Biol.* 433, 227–239. doi: 10.1016/j.ydbio.2017.08.022

Garcia, D. M., Baek, D., Shin, C., Bell, G. W., Grimson, A., & Bartel, D. P. (2011). Weak seed-pairing stability and high target-site abundance decrease the proficiency of lsy-6 and other microRNAs. *Nat. Struct. Mol. Biol.* 18, 1139–1146. doi: 10.1038/nsmb.2115

Grimson, A., Farh, K. K., Johnston, W. K., Garrett-Engele, P., Lim, L. P., & Bartel, D. P. (2007). MicroRNA targeting specificity in mammals: determinants beyond seed pairing. *Mol. cell* 27, 91–105. doi: 10.1016/j.molcel.2007.06.017

Jonkman, J., Brown, C. M., Wright, G. D. *et al.* (2020). Tutorial: guidance for quantitative confocal microscopy. *Nat. Protoc.* 15, 1585–1611. doi: 10.1038/s41596-020-0313-9

Katoh, K., Misawa, K., Kuma, K., & Miyata, T. (2002). MAFFT: a novel method for rapid multiple sequence alignment based on fast Fourier transform. *Nucleic Acids Res.* 30, 3059–3066. doi: 10.1093/nar/gkf436

Liu, W., Xie, Y., Ma, J., Luo, X., Nie, P., Zuo, Z., *et al.* (2015). IBS: an illustrator for the presentation and visualization of biological sequences. *Bioinformatics* 31, 3359–3361. doi: 10.1093/bioinformatics/btv362

Mathews, D.H., Sabina, J., Zucker, M., Turner, H. (1999). Expanded Sequence Dependence of Thermodynamic Parameters Provides Robust Prediction of RNA Secondary Structure. *J. Mol. Biol.* 288, 911-940. doi: 10.1006/jmbi.1999.2700

Matsunami, M., Suzuki, M., Haramoto, Y., Fukui, A., Inoue, T., Yamaguchi, K., *et al.* (2019). A comprehensive reference transcriptome resource for the Iberian ribbed newt *Pleurodeles waltl*, an emerging model for developmental and regeneration biology. *DNA Res.* 26, 217–229. doi: 10.1093/dnares/dsz003

Mi, H., Muruganujan, A., Ebert, D., Huang, X., & Thomas, P. D. (2019). PANTHER version 14: more genomes, a new PANTHER GO-slim and improvements in enrichment analysis tools. *Nucleic Acids Res.* 47, D419–D426. doi: 10.1093/nar/gky1038

Mysona, B. A., Segar, S., Hernandez, C., Kim, C., Zhao, J., Mysona, D., & Bollinger, K. E. (2020). QuPath Automated Analysis of Optic Nerve Degeneration in Brown Norway Rats. *Transl. Vis. Sci. Technol.* 9, 22. doi: 10.1167/tvst.9.3.22

Rehmsmeier, M., Steffen, P., Hochsmann, M., & Giegerich, R. (2004). Fast and effective prediction of microRNA/target duplexes. *RNA* 10, 1507–1517. doi: 10.1261/rna.5248604

Schindelin, J., Arganda-Carreras, I., Frise, E., Kaynig, V., Longair, M., Pietzsch, T., *et al.* (2012). Fiji: an open-source platform for biological-image analysis. *Nat. Methods* 9, 676–682. doi: 10.1038/nmeth.2019
