# Supplementary figures and images for: Functional Characterization of the Lin28/let-7 Circuit During Forelimb Regeneration in Ambystoma mexicanum and Its Influence on Metabolic Reprogramming
Source: Front Cell Dev Biol. 2020 Nov 19;8:562940. doi: 10.3389/fcell.2020.562940 (PMC7710800; doi:10.3389/fcell.2020.562940)

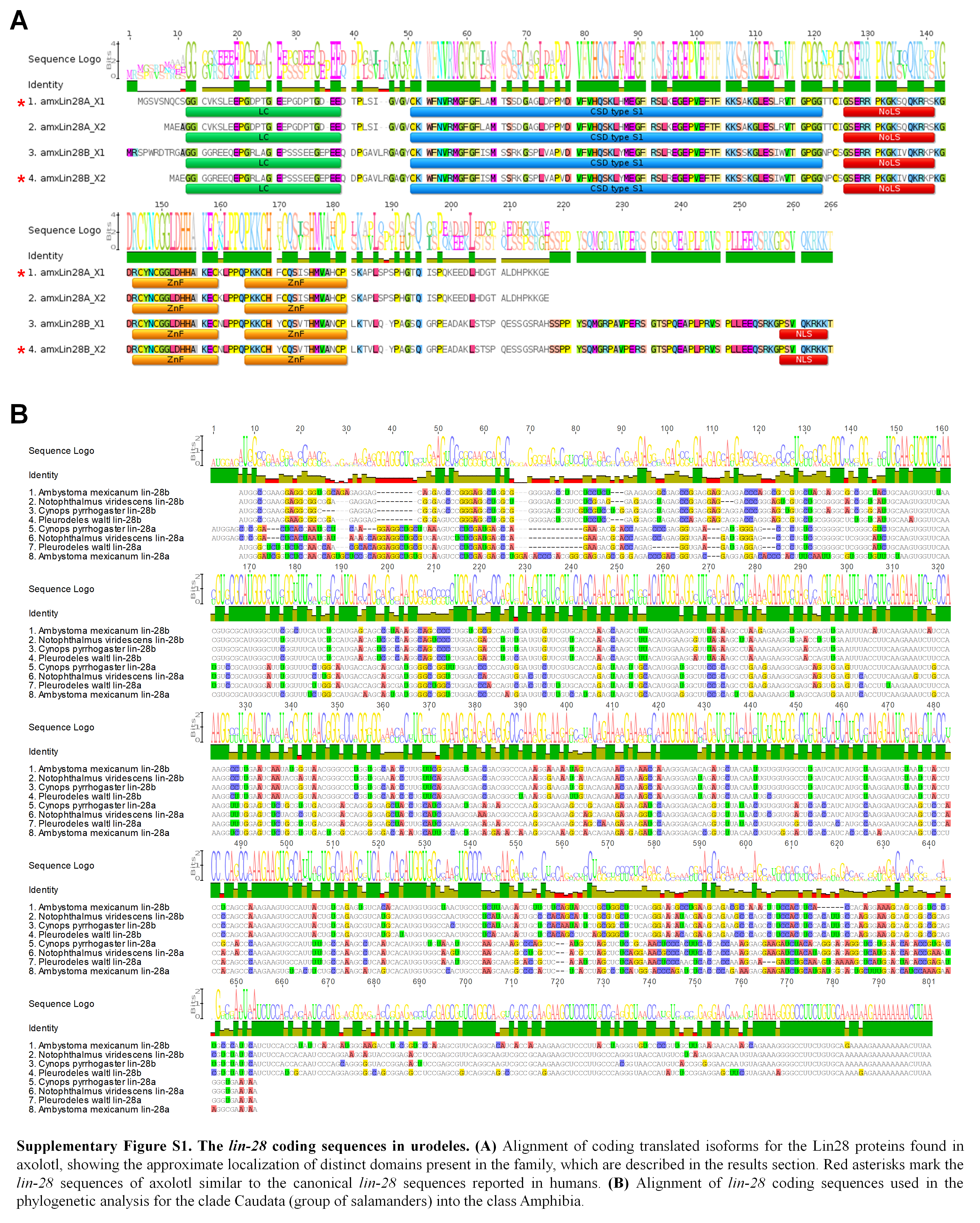

Supplement: Supplementary file 3 [file Image_1.TIF]

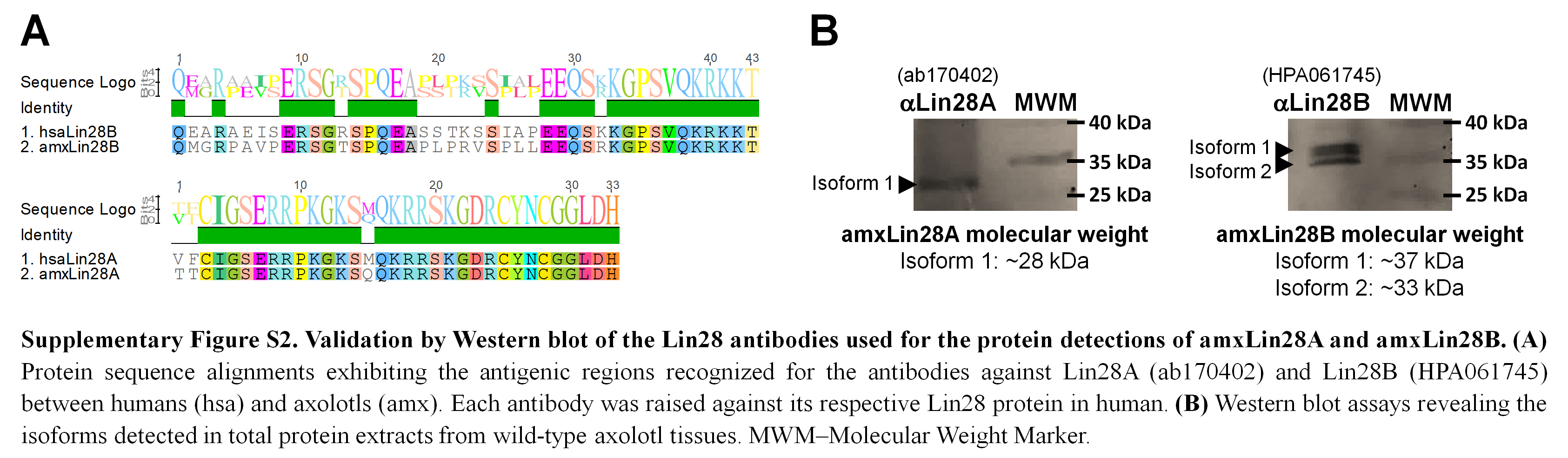

Supplement: Supplementary file 4 [file Image_2.TIF]

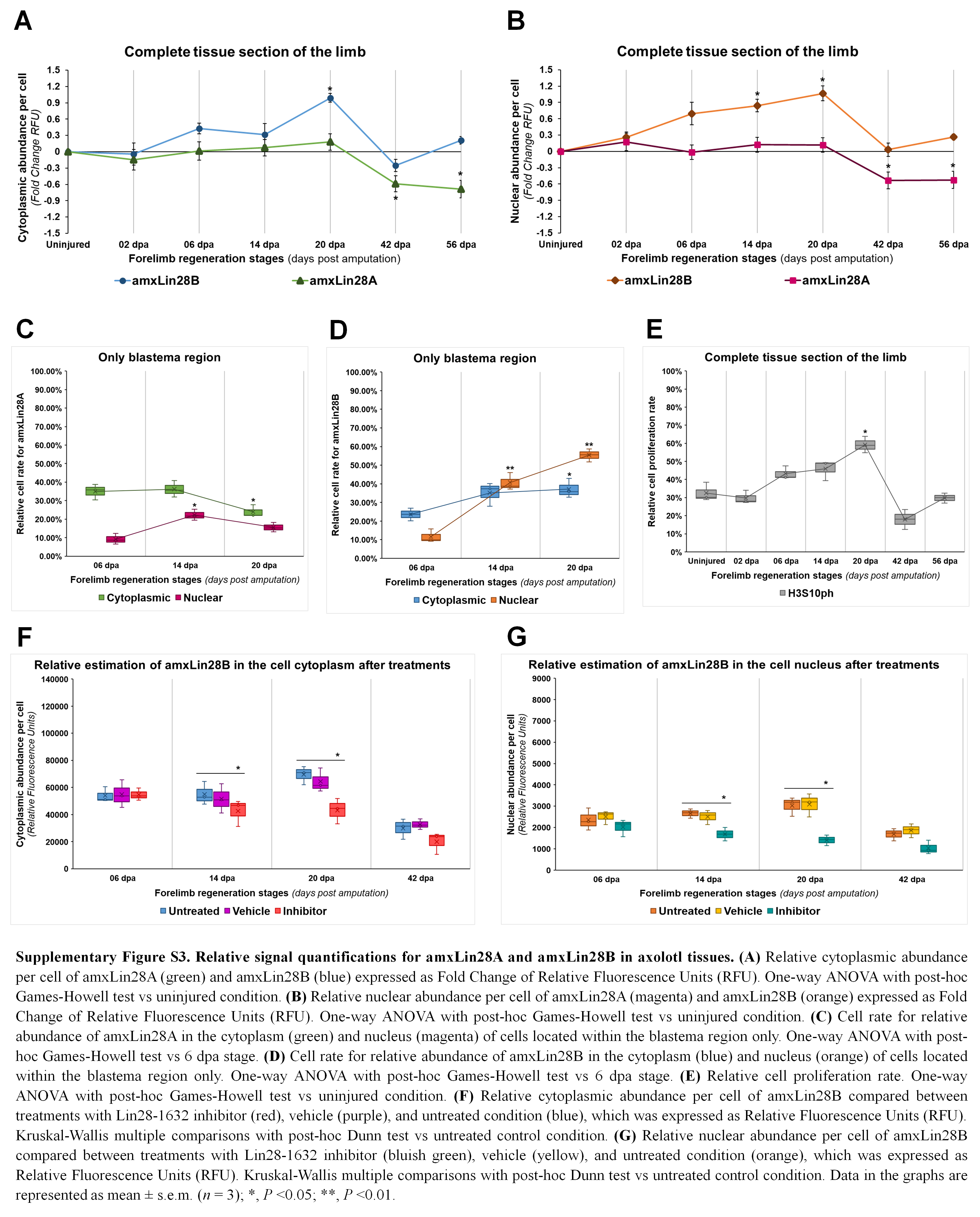

Supplement: Supplementary file 5 [file Image_3.TIF]

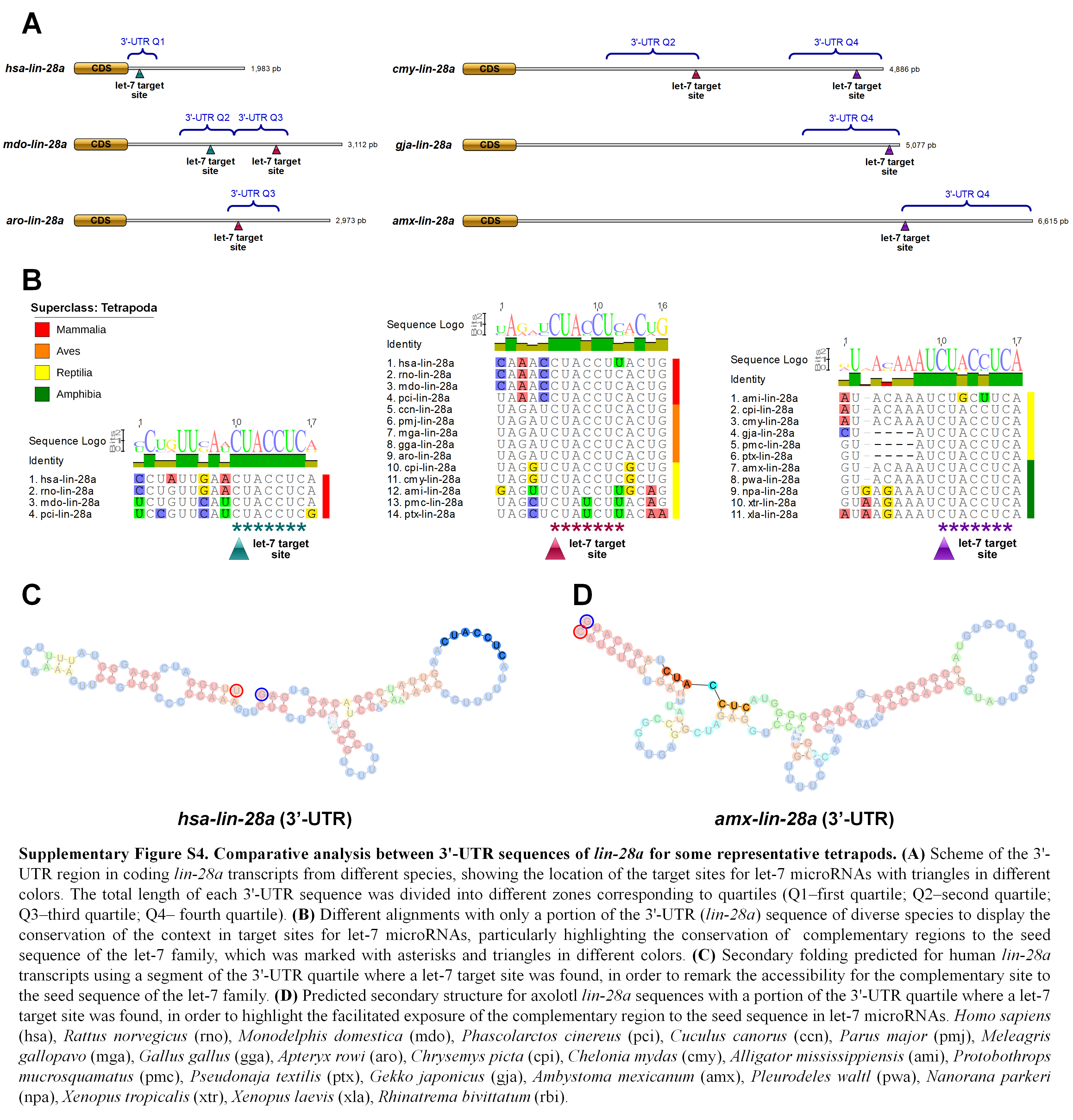

Supplement: Supplementary file 6 [file Image_4.TIF]

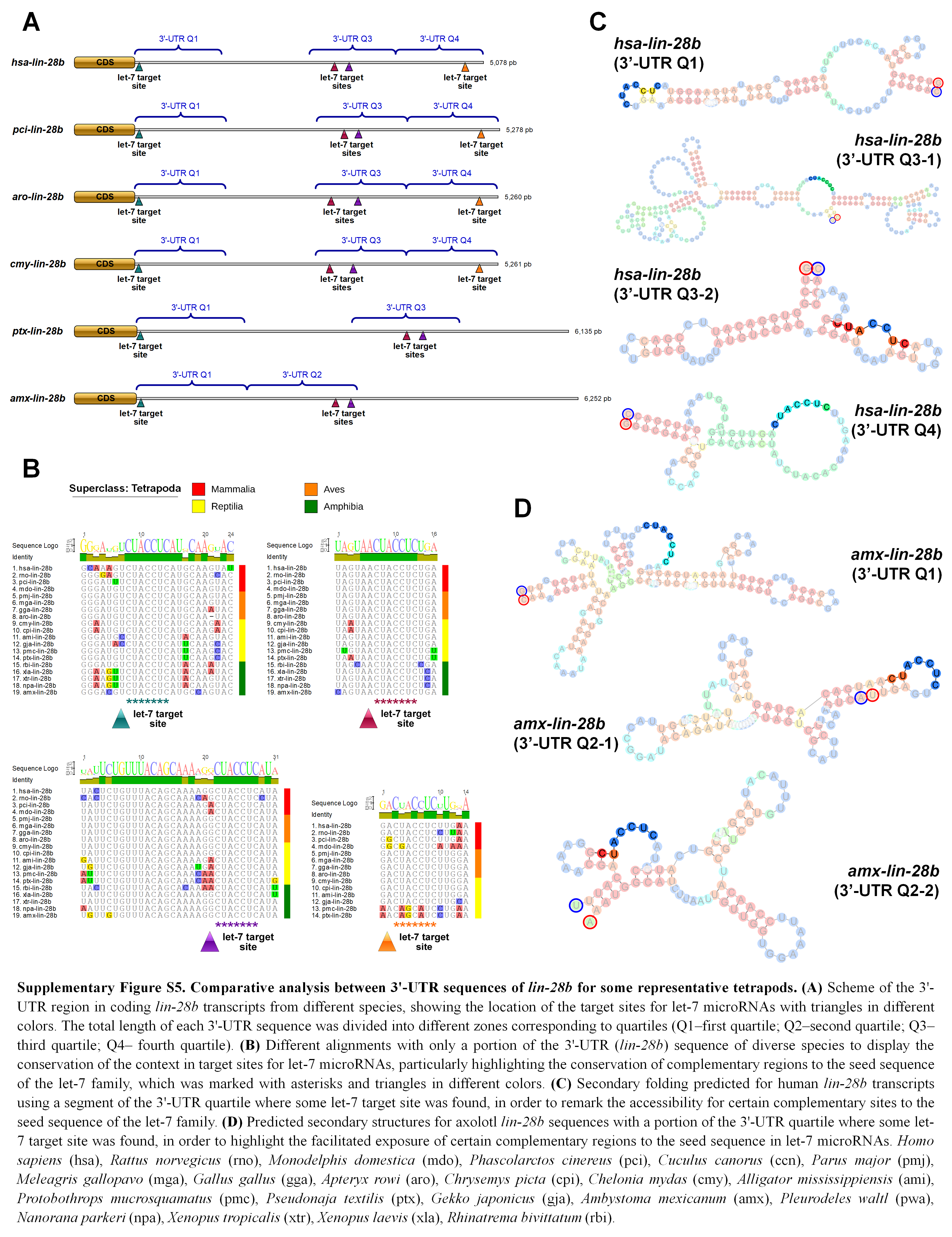

Supplement: Supplementary file 7 [file Image_5.TIF]
